# Predictive value of TRIB3 combined with BMPR2 for major adverse cardiovascular events in elderly coronary heart disease patients undergoing percutaneous coronary intervention

**DOI:** 10.5937/jomb0-56983

**Published:** 2026-01-06

**Authors:** Qiang Zhang, Aiqiao Dong, Tian Wang, Fei Kang, Huan Wang, Jing Sun

**Affiliations:** 1 Xi'an Qinhuang Hospital, Department of Cardiovascular, Shaanxi, 710600, China; 2 Shaanxi Provincial People's Hospital, Department of Otolaryngology, Shaanxi, 701154, China; 3 Shannxi Province Hospital of People's Armed Police Force, Department of Cardiovascular, Xi'an, Shaanxi, 710054, China

**Keywords:** coronary heart disease, cardiovascular diseases, major adverse cardiac events, BMPR2, TRIB3, percutaneous coronary intervention, koronarna bolest srca, kardiovaskularne bolesti, veliki neželjeni kardiovaskularni događaji, BMPR2, TRIB3, perkutana koronarna intervencija

## Abstract

**Background:**

This research aims to explore the correlation of Tribbles Pseudo kinase 3 (TRIB3) and bone morphogenetic protein receptor type 2 (BMPR2) with coronary heart disease (CHD), as well as the evaluation value of the combined detection of the two for major adverse cardiovascular events (MACE) following percutaneous coronary intervention (PCI).

**Methods:**

The study enrolled 152 CHD patients (CHD group) who underwent PCI treatment between January 2023 and May 2024 and 136 healthy individuals (control group) who concurrently underwent physical examination in our hospital. The expressions of TRIB3 and BMPR2 in the serum of both groups were measured. The clinical implications of these two factors in CHD and their diagnostic value for CHD were then analysed. Subsequently, the CHD patients were subjected to a 6-month follow-up. During this period, the occurrence of MACE was recorded, and the evaluation value of the combined detection of TRIB3 and BMPR2 for MACE was analysed.

**Results:**

In the CHD group, the concentration of TRIB3 was significantly elevated compared to the control group, with a notable decline in TRIB3 levels after treatment (P&lt; 0.05). In contrast, the level of BMPR2 in the CHD group was significantly lower than that of the control group, and it increased substantially following treatment (P&lt; 0.05). In the CHD group, TRIB3 and BMPR2 were closely correlated with cardiac troponin I (cTnI) and left ventricular ejection fraction (LVEF) (P&lt; 0.05). The combined detection of TRIB3 and BMPR2 had a diagnostic sensitivity of 76.32% and a specificity of 91.18% for CHD (P&lt; 0.05). The follow-up results showed that 25 patients experienced MACE. The diagnostic sensitivity and specificity of the combined detection of TRIB3 and BMPR2 for MACE were 60.00% and 90.55% , respectively (P&lt; 0.05).

**Conclusions:**

TRIB3 and BMPR2 demonstrated excellent evaluation effects on CHD and the incidence of MACE after PCI.

## Abstract (sr)

**Metode:**

U studiju je uključeno 152 pacijenta sa CHD (CHD grupa) koji su prošli PCI tretman između januara 2023. i maja 2024. godine, kao i 136 zdravih osoba (kontrolna grupa) koje su u istom periodu obavile sistematski pregled u našoj bolnici. Izražaj TRIB3 i BMPR2 u serumu obe grupe je meren. Analizirana je klinička uloga ova dva faktora u KBS i njihova dijagnostička vrednost za CHD. Nakon toga, pacijenti sa CHD su praćeni tokom šest meseci, a u tom periodu su zabeleženi slučajevi MACE. Takođe je analizirana prognostička vrednost kombinovane detekcije TRIB3 i BMPR2 za MACE.

**Rezultati:**

U CHD grupi koncentracija TRIB3 je bila značajno povećana u poređenju sa kontrolnom grupom, dok je nakon lečenja zabeležen značajan pad nivoa TRIB3 (P &lt; 0,05). Nasuprot tome, nivo BMPR2 u CHD grupi bio je značajno niži nego u kontrolnoj grupi, ali je nakon lečenja značajno porastao (P &lt; 0,05). U CHD grupi, TRIB3 i BMPR2 su pokazali značajnu korelaciju sa srčanim troponinom I (cTnI) i frakcijom izbacivanja leve komore (LVEF) (P&lt; 0,05). Kombinovana detekcija TRIB3 i BMPR2 imala je dijagnostičku osetljivost od 76,32% i specifičnost od 91,18% za CHD (P&lt; 0,05). Tokom praćenja, 25 pacijenata je doživelo MACE. Dijagnostička osetljivost i specifičnost kombinovane detekcije TRIB3 i BMPR2 za MACE su iznosile 60,00% i 90,55% , respektivno (P&lt; 0,05).

**Zaključak:**

TRIB3 i BMPR2 pokazali su odlične proceniteljske efekte u otkrivanju CHD i predviđanju učestalosti MACE nakon PCI.

## Introduction

Coronary heart disease (CHD) represents one of the most prevalent cardiovascular diseases among middle-aged and elderly people, and it stands as the most common form of heart disease [1]. A study by Abdelatif N et al. [2] revealed that the average incidence rate of CHD approximated 1.29% from 1990 to 2017. Arroyo-Quiroz C [3] showed that from 2000 to 2012, CHD mortality increased by 33.8% in men and 22.8% in women. Pathophysiologically, CHD is characterised by the stenosis or occlusion of the arterial walls caused by plaques formed by the accumulation of cholesterol and other substances in the coronary arteries, leading to myocardial ischemia, hypoxia and, in severe cases, necrosis [4]. Percutaneous coronary intervention (PCI) is the most direct and effective treatment modality in clinical treatment. Despite its well-established clinical efficacy, the invasive nature of PCI procedures often engenders a spectrum of adverse postoperative sequelae. Major adverse cardiovascular events (MACE) are prevalent [5]. As reported in a statistical analysis by Madhavan MV et al., [6] among 25,032 CHD patients who underwent PCI, the incidence of MACE within the first year of follow-up peaked at 17.9%. Currently, MACE has been clinically identified as a crucial determinant influencing the prognosis and recovery of CHD patients. Thus, developing effective strategies to safeguard against MACE has become a focal point of intense clinical investigation [7].

Currently, the medical community actively endeavours to evaluate the risk of MACE through objective diagnostic markers. For instance, Wang L et al. comprehensively analysed the efficacy of the triglyceride glucose (TyG) index combined with the neutrophil-to-lymphocyte ratio (NLR) in predicting MACE [8]. Meanwhile, Huo Y posited that the Th1/Th2 ratio, Th17/Treg ratio and the occurrence of MACE in CHD patients were related [9]. These research efforts have undeniably provided valuable insights. Nevertheless, the absence of highly specific markers has left substantial room for enhancement in the early-stage assessment of MACE. Tribbles Pseudo kinase 3 (TRIB3), a member of the pseudo kinase family, has been extensively studied in metabolic diseases, cancer, cardiovascular diseases, etc. TRIB3 is activated in the presence of pronounced hypoxia, endoplasmic reticulum stress, and oxidative stress in the body [10]. Intriguingly, these physiological responses align with the underlying pathological mechanisms of MACE following PCI [11].

Regarding CHD, the study by Prudente S et al. [12], mentioned that TRIB3 is a highly sensitive regulatory coding gene in coronary artery disease, which lays the foundation for TRIB3 to be used as a specific diagnostic indicator for CHD. Furthermore, the research conducted by Chan MC et al. [13] elucidated that TRIB3 functions as a positive modulator of the bone morphogenetic proteins (BMP) signalling path way. It exerts its influence by mediating the transduction process of bone morphogenetic protein receptor type 2 (BMPR2), consequently playing a role in regulating blood vessel synthesis. Earlier investigations have demonstrated that BMPR2 exhibits anti-inflammatory and anti-atherosclerotic properties in endothelial cells [14]. The gene transduction cascade involving TRIB3 and BMPR2 appears to be intricately associated with the progression of CHD. As such, this pair of molecules holds significant promise as potential markers for assessing the risk of MACE.

Notwithstanding, to date, no published studies have been able to corroborate this hypothesis. Therefore, in this study, we will observe the profiles of TRIB3 and BMPR2 in CHD and, through a combined-detection approach, analyse the value of the two in assessing MACE after PCI. These findings will provide new references and guidelines for future clinical use in evaluating CHD and MACE, thus better safeguarding the prognostic health of patients.

## Materials and methods

### Study design and participants

This investigation was a retrospective analysis. First, we calculated the sample size needed for this study: n=Z^2^x[P x(1-P)]/E^2^. statistic (Z)=1.96, error (E)=10%, probability (P)=0.5, sample size (N)=96. After obtaining approval from the Ethics Committee of our hospital (No. kl202309), 152 CHD patients (CHD group) who underwent PCI in our hospital between January 2023 and May 2024 were recruited. Additionally, 136 healthy individuals who underwent routine health examinations from the same time frame served as the control group. All participants were fully informed about the nature of this study and provided their written informed consent. The study was conducted strictly with the principles of the Declaration of Helsinki; all study subjects signed an informed consent form.

Inclusion criteria: (1) Participants were required to be 65 or older. (2) A definitive diagnosis of CHD had to be established via coronary angiography, with the identification of 1-3 diseased coronary vessels. (3) Patients were required to meet the well-established clinical indications for PCI, having undergone the implantation of 1-3 stents. (4) Complete clinical, laboratory, and follow-up data were essential for inclusion. 5) No acute myocardial infarction or unstable angina within the last 3 months.

Exclusion criteria: (1) Individuals with concurrent liver or kidney dysfunction were excluded. (2) Participants diagnosed with autoimmune diseases were not included. (3) Those with a previous history of PCI treatment were ineligible. (4) Patients with cerebrovascular diseases were excluded. (5) Cases complicated by myocarditis, valvular heart disease, or other significant cardiac conditions were excluded. 6) Patients with combined tumors.

### Data collection

We collected the levels of TRIB3 and BMPR2 detected at the time of admission for all study subjects. Additionally, the levels of TRIB3 and BMPRII for patients with CHD were obtained on the day following PCI. The detailed protocol entailed collecting 4 mL of fasting venous blood from patients into clot-promoting tubes. The samples were then allowed to stand for 30-60 minutes to ensure complete blood coagulation, after which centrifugation was performed. The supernatant serum was carefully separated for subsequent Enzyme-Linked Immunosorbent Assay (ELISA) analysis. The ELISA kit was purchased from Shanghai Yanqi Biotechnology Co., Ltd., and the operation was carried out strictly following the kit instructions. In addition, the routine examination items of CHD patients upon admission were also collected, specifically including Left ventricular ejection fraction (LVEF), cardiac troponin I (cTnI), and creatine kinase-MB (CK-MB). LVEF was obtained by cardiac ultrasound (ACUSONX300, Siemens); cTnI and CK-MB were obtained using a fully automated electrochemiluminescence analyser (Myriad, CL-1000i).

### Follow-up survey

All CHD patients were subjected to a six-month follow-up investigation after the completion of PCI treatment. The follow-up was carried out in the form of hospital reviews, with a monthly frequency. It was recorded whether the patient had undergone MACE during this procedure. Diagnostic criteria: (1) Cardiovascular death, (2) Non-fatal myocardial infarction, (3) Non-fatal stroke, (4) Ischemia-driven revascularisation, (5) Hospitalisation for heart failure, (6) Unstable angina or arrhythmia [15].

### Observation indicators

The expression profiles of TRIB3 and BMPR2 in CHD and their diagnostic value for CHD were analysed.

The correlations of TRIB3 and BMPR2 with LEVF, cTnI, and CK-MB in CHD patients were explored.

The value of TRIB3 and BMPR2 in assessing MACE was determined.

### Statistical analysis

The data in this study were processed and analysed using SPSS 25.0 software. Measurement data were first subjected to the Shapiro-Wilk normality test. Data conforming to a normal distribution were expressed as mean ± standard deviation (x̄±s). For comparisons between groups, an independent-sample t-test was employed, while a paired t-test was used for within-group comparisons. Enumeration data were presented as percentages [n(%)], and comparisons were made using the χ^2^ test or Fisher's exact test. To explore the relationships between variables, the Pearson correlation coefficient was employed for correlation analysis. Logistic regression analysis was carried out to identify and analyse the influencing factors. Moreover, the receiver operating characteristic (ROC) curve analysis was performed to comprehensively evaluate the diagnostic value of relevant indicators. The effectiveness of the diagnosis was judged by the area under the curve (AUC), with an AUC closer to 1 indicating a better diagnosis. Based on the logistic regression analysis results, the joint formula Log(P)=constant + regression coefficient × TRIB3 + regression coefficient × BMPR2 was calculated, followed by ROC curve analysis. When P<0.05 indicates that the difference is statistically significant.

## Results

### General information about the CHD group and the control group

Regarding general parameters such as age, gender, and body mass index (BMI), no significant differences were observed between the CHD group and the control group (P>0.05). However, in terms of cardiac function, LVEF was lower in the CHD group than in the control group, while cTnI and CK-MB were higher than in the control group (P<0.0 5; Table 1).

**Table 1:** Comparison of general information. Body mass index (BMI), coronary heart disease (CHD), left ventricular ejection fraction (LVEF), cardiac troponin I (cTnI), and creatine kinase-MB (CK-MB).

	Control group	CHD group	t (χ^2^)	P
n	136	152		
Age	70.82±3.88	70.82±4.08	0.013	0990
Male/female	76/60	94/58	1.054	0305
BMI (kg/m^2^)	24.74±2.35	25.02±2.40	0.989	0.324
Smoking history	40 (29.41)	51 (33.55)	0.569	0.451
Diabetes mellitus	22 (16.18)	34 (22.37)	1.757	0185
Hypertension	27 (19.85)	42 (27.63)	2.384	0.123
Hyperlipidemia	19 (13.97)	32 (21.05)	2.470	0.116
Family history of CHD	10 (7.35)	19 (12.50)	2.100	0.147
Number of diseased vessels (1/2/3)	-	46/72/34	-	-
LVEF (%)	62.97±6.74	33.98±5.77	39.354	<0.001
cTnI (ng/mL)	0.03±0.01	2.89±0.72	46.437	<0.001
CK-MB (U/L)	10.35±3.88	19.98±5.02	18.042	<0.001

### Clinical significance of TRIB3 and BMPR2 in CHD

**Figure 1:**
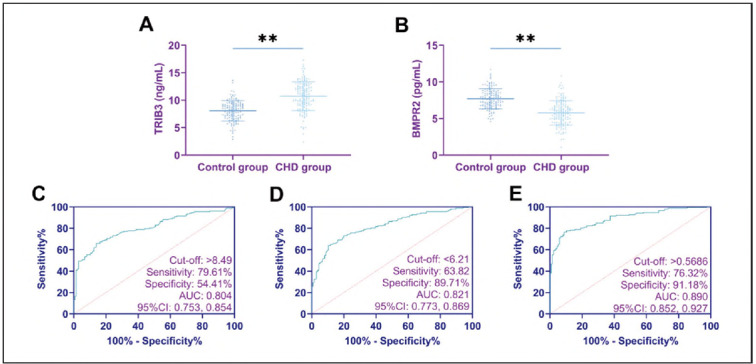
Expression and diagnostic significance of TRIB3 and BMPR2 in CHD.

Compared with the control group, the CHD group demonstrated an elevation in TRIB3 levels and a reduction in BMPR2 levels (P<0.01). The ROC curve analysis indicated that TRIB3 and BMPR2 exhibited outstanding diagnostic performance for CHD. Logistic regression analysis showed that TRIB3 was an independent risk factor for CHD, whereas BMPR2 acted as a protective factor (P< 0.001). When the two were combined for diagnosing CHD, the sensitivity and specificity reached 76.32% and 91.18%, respectively (P<0.05; Figure 1 and Table 2).

**Table 2:** Effect of TRIB3, BMPR2 on CHD. Regression coefficient (B), standard error (S.E.), odds ratio (OR), 95% confidence interval (95%CI).

	B	S.E.	Wald χ^2^	P	OR	95%CI
TRIB3	0.534	0.082	41.979	<0.001	1.705	1.451, 2.004
BMPR2	-0.888	0.129	47.153	<0.001	0.411	0.319, 0.530
Constant	1.177	1.012	1.352	0.245	3.244	-

### Association of TRIB3 and BMPR2 with patholog ical features of CHD

**Figure 2:**
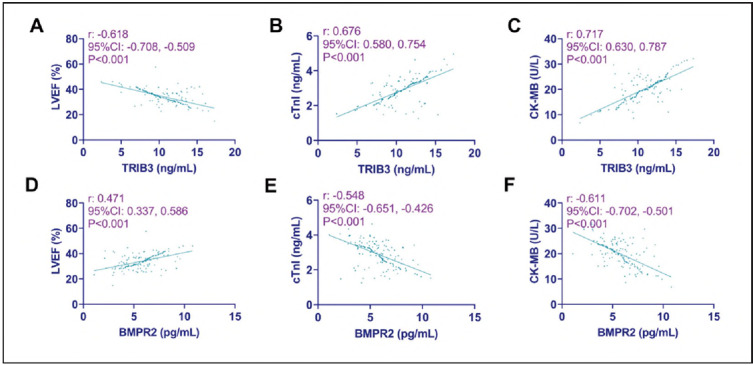
Relationship between TRIB3, BMPR2 and cardiac function in CHD patients.

Pearson correlation coefficient analysis showed that TRIB3 in CHD patients was negatively correlated with LVEF and positively correlated with cTnI and CK-MB (P<0.001). The results were reversed for BMPR2, i.e., BMPR2 was positively correlated with LVEF and negatively correlated with cTnI and CK-MB (P< 0.05; Figure 2).

### Changes in TRIB3 and BMPR2 before and after treatment

**Figure 3:**
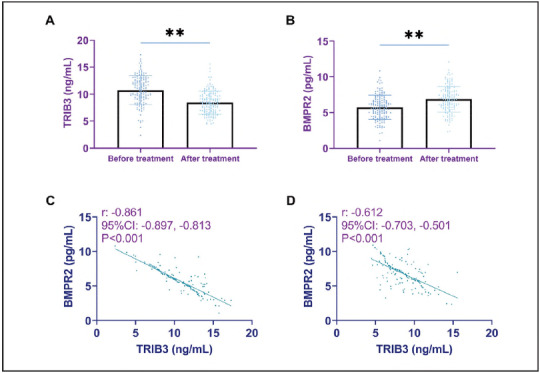
Changes in TRIB3 and BMPR2 before and after treatment: TRIB3 was reduced, and BMPR2 was elevated after treatment.

After treatment, a decrease in TRIB3 levels was observed in the CHD group, accompanied by an increase in BMPR2 levels (P<0.001). Analysis using the Pearson correlation coefficient revealed a notable negative correlation between TRIB3 and BMPR2 among CHD patients, regardless of whether the assessment was conducted before or after treatment (P<0.001; Figure 3).

### Assessment of the predictive value of TRIB3 and BMPR2 for MACE

**Figure 4:**
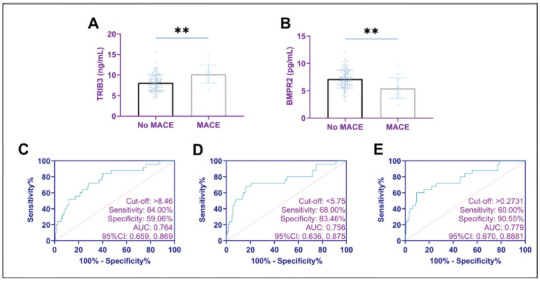
Expression of TRIB3 and BMPR2 in MACE and diagnostic significance.

Results from the follow-up investigation indicated that MACE occurred in 25 patients, accounting for an overall incidence rate of 16.45% (25/152). Compared to patients who did not experience MACE, those with MACE exhibited significantly higher post treatment TRIB3 levels and lower post-treatment BMPR2 levels (P<0.01). Using ROC curves, we found that the sensitivity and specificity of the combined TRIB3 and BMPR2 assay for assessing MACE occurrence were 60.00% and 90.55%, respectively (P<0.05; Figure 4 and Table 3).

**Table 3:** Effect of TRIB3, BMPR2 on CHD.

	B	S.E.	Wald χ^2^	P	OR	95%CI
TRIB3	0.291	0.135	4.684	0.030	1.338	1.028, 1.742
BMPR2	-0.439	0.185	5.615	0.018	0.645	0.449, 0.927
Constant	-1.523	2.042	0.556	0.456	0.218	-

## Discussion

In this research, we have identified the abnormal expression profiles of TRIB3 and BMPR2 in CHD. Moreover, our findings have demonstrated the exceptional capacity of these two factors to evaluate the occurrence of CHD and the incidence of MACE following PCI. The results of this study provide novel and valuable guidance for future clinical practice.

TRIB3 is located on human chromosome 20p13-p12.2. Initial research discovered that pseudo-kinase and MEKs domains could bind to various transcription factors and signaling proteins, inhibiting relevant regulatory pathways [16]. More recently, an increasing number of studies have indicated that TRIB3 also plays a crucial role in regulating cardiovascular diseases and is involved in the progression of atherosclerosis [17]. In the present study, the elevated expression of TRIB3 in CHD serves to reaffirm this perspective. Moreover, TRIB3 has been found to exhibit a remarkable correlation with markers such as cTnI and LVEF in CHD patients, indicating the close association between TRIB3 and the progression of CHD. As noted in the study by Rines AK et al. [18], rapamycin-induced autophagy and the overexpression of TRIB3 can give rise to aberrant cardiac function and the generation of myocardial free radicals in murine models; conversely, the silencing of TRIB3 expression can achieve a cardioprotective effect. This suggests that TRIB3 holds significant promise as a potential therapeutic target for endoplasmic reticulum stress-mediated cardiac function impairment. In the present study, a reduction in TRIB3 levels was observed in CHD patients following treatment, consistent with the scenario above. Furthermore, the ROC curve demonstrated that TRIB3 exhibited remarkable discriminative ability in evaluating both the occurrence of CHD and MACE, underscoring the potential of TRIB3 as an indicator for assessing the progression and severity of CHD. Recently, Mannino GC et al. [19], reported that the upregulation of TRIB3 expression plays a pathogenic role in left ventricular mass further corroborating our viewpoint.

However, as mentioned above, since current research on TRIB3 is not solely confined to cardiovascular diseases, relying solely on TRIB3 to judge the condition of CHD may be bereft of the requisite precision and comprehensiveness. In light of this, the strategy we proffer entails a combined diagnostic approach, leveraging BMPR2, a key component in the downstream transduction pathway of TRIB3. As a member of the BMP family, current research on BMPR2 mostly focuses on pulmonary arterial hypertension [20] [21]. In contrast, studies that directly relate to CHD remain relatively scarce. Therefore, we first observed the differences in BMPR2 expression between the two groups of research subjects. It was found that BMPR2 was downregulated in CHD and exhibited an upregulation following therapeutic inter ventions, showing an expression change trend utterly opposite to that of TRIB3. This initially corroborates the protective effect of BMPR2 in CHD, which is con sistent with the viewpoints of previous research [22]. A single-cell RNA sequencing study by Du M et al. [23] indicated that BMPR2 gene mutations regulate right ventricular function, highlighting the relationship between BMPR2 and maintaining cardiac functional integrity. Consequently, in the present study, the analysis revealed that while the diagnostic sensitivity of BMPR2 for CHD and MACE was not as significant as that of TRIB3, there was a notable and substantial enhancement in the diagnostic specificity of BMPR2. This finding is of great importance, as it establishes a robust and reliable basis for developing a combined detection protocol integrating TRIB3 and BMPR2. The marked improvement in diagnostic efficacy underscores the substantial potential of this combined diagnostic approach, which can provide more reliable safeguards for future clinical management of CHD and contribute to enhanced patient care and improved therapeutic outcomes.

Nevertheless, given that the present study is a single-centre retrospective analysis, the limited sample size represents a substantial constraint. In future investigations, it is imperative to substantially increase the number of cases to corroborate further the evaluative applications of TRIB3 and BMPR2 in CHD. Additionally, the absence of in-vitro experimental support precludes our ability to elucidate the precise mechanistic roles of TRIB3 and BMPR2 in CHD. In light of this deficiency, we are committed to supplementing a more comprehensive set of experiments, thus enhancing the accuracy and reliability of the results presented in this paper. To rapidly assess MACE, we used blood samples from the 2nd d after PCI for analysis, which may result in TRIB3 and BMPR2 assay results being affected by surgical stress. Therefore, we should add TRIB3 and BMPR2 examinations at multiple time points in subsequent studies to perform dynamic analysis.

## Conclusion

TRIB3 and BMPR2 are potentially evaluable in CHD and MACE development after PCI. These findings can offer reliable safety guidance for future clinical management of CHD, thereby safeguarding patients' long-term health and prognosis.

## Dodatak

### Data sharing statement

All data and materials are available from the corresponding author.

### Conflict of interest statement

All the authors declare that they have no conflict of interest in this work.
